# Diabetes and artificial intelligence beyond the closed loop: a review of the landscape, promise and challenges

**DOI:** 10.1007/s00125-023-06038-8

**Published:** 2023-11-18

**Authors:** Scott C. Mackenzie, Chris A. R. Sainsbury, Deborah J. Wake

**Affiliations:** 1 Population Health and Genomics, School of Medicine, University of Dundee, Dundee, UK; 2 Institute for Applied Health Research, University of Birmingham, Birmingham, UK; 3 Usher Institute, The University of Edinburgh, Edinburgh, UK; 4 Edinburgh Centre for Endocrinology and Diabetes, NHS Lothian, Edinburgh, UK

**Keywords:** Artificial intelligence, Barriers, Big data, Clinical informatics, Decision support, Diabetes, Facilitators, Machine learning, Personalised medicine, Review, Type 2 diabetes

## Abstract

The discourse amongst diabetes specialists and academics regarding technology and artificial intelligence (AI) typically centres around the 10% of people with diabetes who have type 1 diabetes, focusing on glucose sensors, insulin pumps and, increasingly, closed-loop systems. This focus is reflected in conference topics, strategy documents, technology appraisals and funding streams. What is often overlooked is the wider application of data and AI, as demonstrated through published literature and emerging marketplace products, that offers promising avenues for enhanced clinical care, health-service efficiency and cost-effectiveness. This review provides an overview of AI techniques and explores the use and potential of AI and data-driven systems in a broad context, covering all diabetes types, encompassing: (1) patient education and self-management; (2) clinical decision support systems and predictive analytics, including diagnostic support, treatment and screening advice, complications prediction; and (3) the use of multimodal data, such as imaging or genetic data. The review provides a perspective on how data- and AI-driven systems could transform diabetes care in the coming years and how they could be integrated into daily clinical practice. We discuss evidence for benefits and potential harms, and consider existing barriers to scalable adoption, including challenges related to data availability and exchange, health inequality, clinician hesitancy and regulation. Stakeholders, including clinicians, academics, commissioners, policymakers and those with lived experience, must proactively collaborate to realise the potential benefits that AI-supported diabetes care could bring, whilst mitigating risk and navigating the challenges along the way.

**Graphical Abstract:**

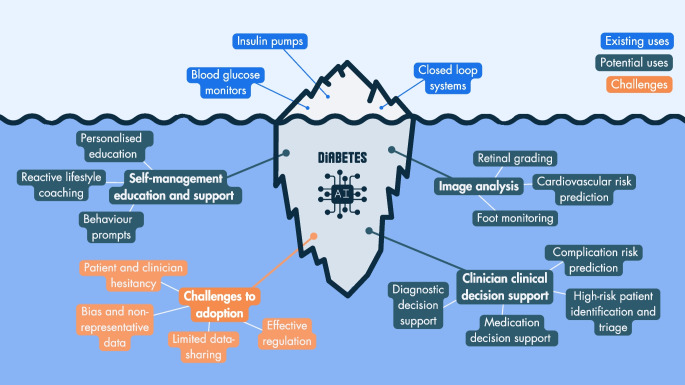

**Supplementary Information:**

The online version of this article (10.1007/s00125-023-06038-8) contains a slideset of the figures for download, which is available to authorised users.


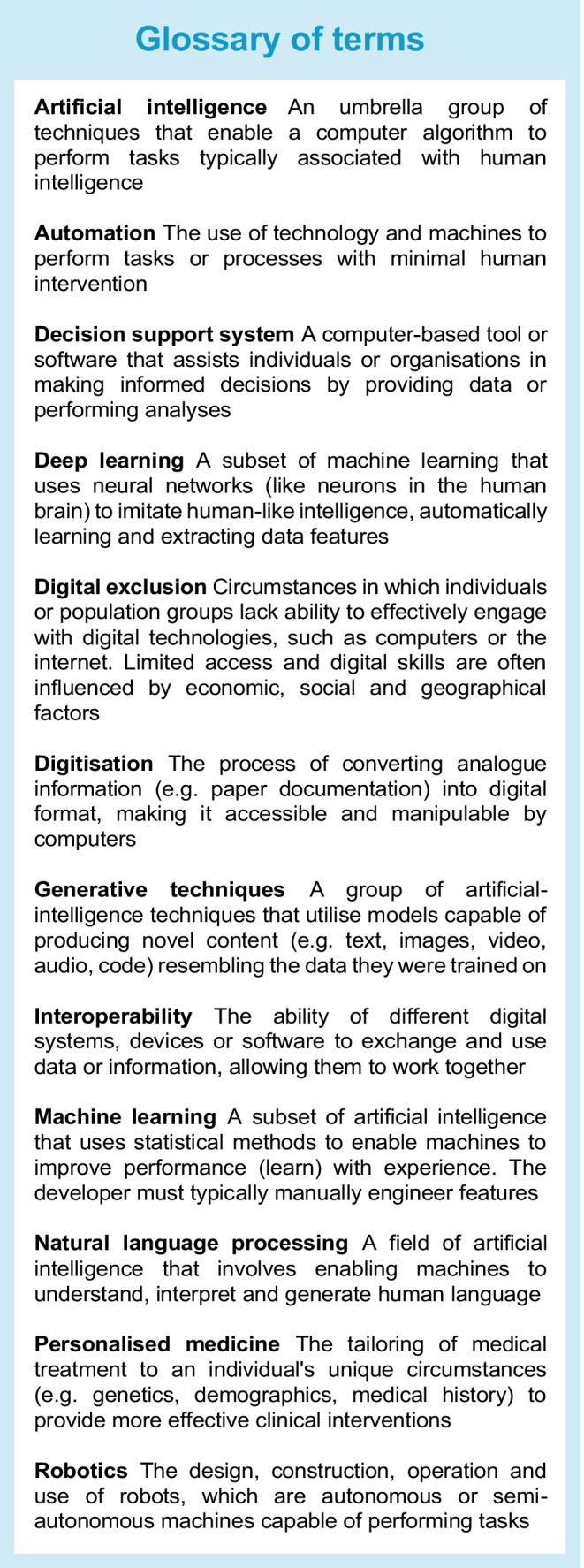


## Introduction

The rapid growth in diabetes prevalence constitutes one of the greatest global health emergencies of the 21st century. Currently, diabetes affects 10% of the worldwide population, accounting for almost 1 trillion US dollars in expenditure. Management and sequalae of largely preventable complications consume most of the cost [1]. As the prevalence of diabetes grows, current models of diabetes care will be unable to scale to meet demand. Thus, the need for more efficient and cost-effective management is pressing. By facilitating personalised and streamlined care, data-driven solutions could improve patient outcomes and reduce the burden on healthcare providers. As such, they offer a promising opportunity to facilitate improved cost-effective care at scale worldwide.

### The potential for data-driven diabetes care

Data-driven artificial-intelligence (AI) solutions are widely used in many areas of life, such as banking and travel, but healthcare has been slow to adopt them. Diabetes care lends itself well to the application of data-driven approaches, given the clear evidence-based targets and guidelines, as well as the high prevalence, necessitating scalable cost-effective healthcare delivery. Adaptive AI approaches can support clinical practice where there is emerging complexity, such as in diagnostic subtyping and personalised management (as highlighted in the 2020 ADA/EASD precision medicine Consensus Report [2]). The wide range of patient-facing digital applications to support aspects of self-management (including glucose control, dietary choice, activity tracking, foot screening and home diagnostics) are beginning to deliver a paradigm shift towards patient-led (and patient-empowerment in) healthcare. AI approaches have the potential to deliver insights directly to patients in a way that may influence their behaviour and downstream outcomes. Despite this, AI-based technology is not yet being implemented or adopted at scale for day-to-day diabetes management, and awareness is limited amongst the clinical workforce, with the exception being the use of diabetes technology in type 1 diabetes (i.e. insulin pumps, glucose sensors and closed-loop systems).

### The data tsunami

Data has immense potential to support diabetes care and can be sourced from many outlets. For instance, medical records can offer information on demographics, medical history, diagnoses, medications, physiological observations, and laboratory and imaging data. People with diabetes can contribute by providing reported data, such as symptom scoring, by completing outcome or experience measure questionnaires. Home-recorded data, including glucose levels, blood pressure, weight, diet and activity, and results from emerging home diagnostics and screening tools, like home blood and urine testing and smart foot insoles, contribute to a broader understanding. Additionally, wider social and environmental data, such as weather, economic trends, consumer habits, food purchases, nutritional intake, peer interaction and geography, can help personalise and localise self-management advice. Whilst genomic data are not yet widely available, the advent of rapid-throughput and low-cost genotyping will undoubtedly make them a vital input for future healthcare-prediction or precision-medicine tools.

The potential of these data is significant, but at present most data are underutilised, as practical tools to aggregate, synthesise, understand and present them in a diabetes context are limited, despite an exponential increase in publications relating to diabetes and AI in recent years [3]. Utilisation of this wealth of data through AI and digitally supported healthcare is increasingly recognised by key strategic, funding and commissioning organisations, such as governments and healthcare providers, with substantial increases in funding and research focused on AI, personalised medicine and digitally enabled healthcare delivery [4, 5].

### What is AI?

**Fig. 1:**
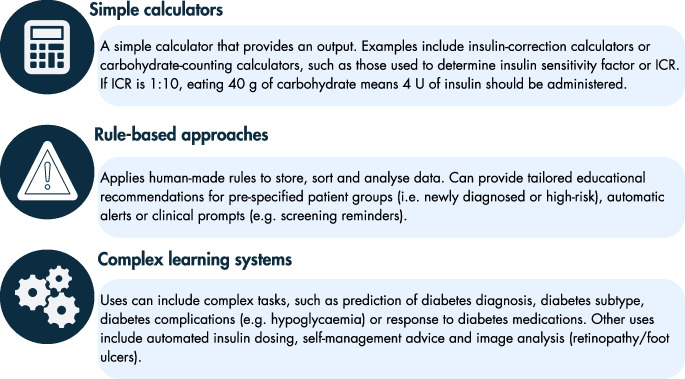
Data-driven tools can include simple calculators, rule-based approaches and AI-driven approaches. Each approach has varied applications in diabetes care. ICR, insulin:carbohydrate ratio. This figure is available as part of a downloadable slideset

AI is an umbrella term that can be defined in many ways. The Encyclopaedia Britannica explains AI as ‘the ability of a digital computer or computer-controlled robot to perform tasks commonly associated with intelligent beings’ [6]. Examples of AI technologies can include automation, robotics, natural language processing, generative techniques (such as image generation) and machine learning (see Text box). Whilst a purist approach would require an element of ‘learning’ within an AI system, and separation of machine learning from AI, a broader definition includes a range from simple automated calculations, through to rule-based approaches and more complex learning systems (see Fig. 1) [3].

**Fig. 2:**
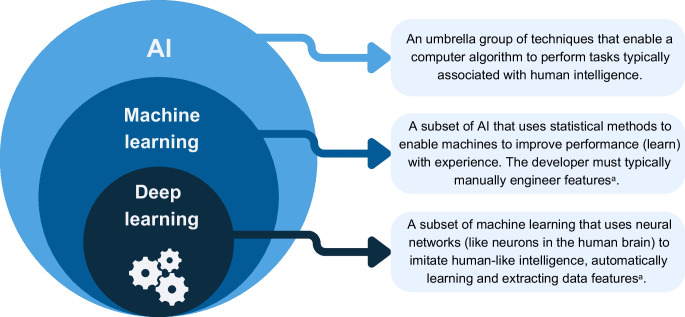
An overview of AI and its subcomponents, including machine learning and deep learning. ^a^A feature refers to an individual and measurable characteristic of data and is typically numerical or categorical. This figure is available as part of a downloadable slideset

Both simple calculators and rule-based approaches are used throughout diabetes care in various contexts, such as for insulin dosing or clinical alerts [7]. Beyond these, more complex systems exist, which commonly involve experiential learning (see Fig. 2). Machine learning can be broadly methodologically categorised into supervised, unsupervised and reinforcement approaches (see Table 1). These techniques have broad applications within diabetes care, such as prediction of diagnosis, complication occurrence or treatment response, image analysis and medication management.

**Table 1:** An overview of machine-learning methodologies (supervised, unsupervised and reinforcement)

	Supervised ML	Unsupervised ML	Reinforcement ML
Definition	Learning with a labelled dataset to find rules that map inputs to outputs	Identification of patterns or features in unlabelled data	Learning through interaction with the environment
Aim	Predict outcomes	Discover underlying patterns	Find an optimal series of actions
Type of data	Labelled	Unlabelled	Dynamic environment
Type of problem	Classification and regression	Association and clustering	Exploration and exploitation
Example algorithms	Decision trees, logistic regression, linear regression, support vector machine, naive Bayes, artificial neural networks	*k*-means clustering, principal component analysis, hierarchical clustering, artificial neural networks	Monte Carlo, SARSA, Q-learning
Example of use in diabetes	Diagnosis or complication prediction, decision support	Retinal image analysis, identification of clinically significant sub-phenotypes	Automated insulin delivery and optimisation of wider therapeutic strategies

ML, machine learning; SARSA, state–action–reward–state–action

Contreras and Vehi categorise AI methodologies into three main groups: (1) methods used for exploring and discovering information; (2) methods aimed at learning to use information; and (3) those used for extracting conclusions from information [3]. However, data collection and analysis alone are not enough to deliver impact; data needs to be transformed into a usable output resulting in a change in practice or behaviour. Further steps can include utilising outputs to deliver continuous improvement within a learning health system, and presenting clinically relevant outputs through a decision support system (DSS) that can integrate with clinical care and aim to modify behaviour [8].

### What is a DSS?

**Fig. 3:**
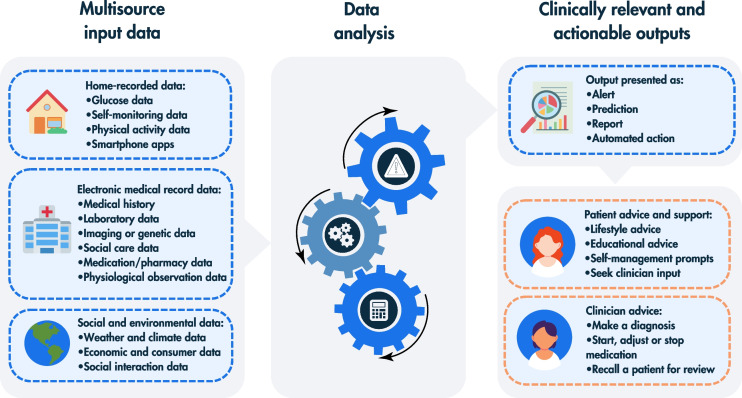
An overview of multisource data inputs (home, hospital and environmental data), analysis techniques (simple calculators, rule-based approaches and AI-based approaches [depicted by the image of the calculator, exclamation mark and cogs, respectively]) and potential clinically relevant and actionable outputs relevant to diabetes care. This figure is available as part of a downloadable slideset

Clinical DSS are key tools that enable delivery of AI into the hands of patients or clinicians. In essence, DSS are computer programs that support analysis of large datasets to deliver timely information, usually at the point of care, with the aim to improve care quality and clinical outcomes. Clinical DSS usually turn data (after application of logic/transformation) into knowledge or meaningful advice through a digital interface (e.g. report, dashboard, alert, message or other output; see Fig. 3).

**Fig. 4:**
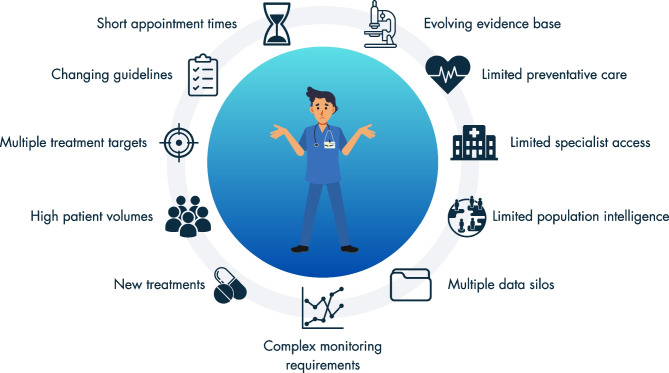
Why is clinician decision support necessary? As shown in the figure, many factors demand clinician time and drive the complexity of diabetes care. Clinician DSS aim to improve efficiency and reduce cognitive burden through prompts, automation and targeted advice. Targeted and timely DSS can make up-to-date, evidence-based information (e.g. guidelines, treatments, monitoring requirements) available to clinicians to facilitate informed decision-making. Additionally, through the analysis and synthesis of diverse data sources, DSS can support streamlined and efficient care, which is crucial to address challenges like limited appointment times and high patient volumes. This figure is available as part of a downloadable slideset

For clinicians, data-driven AI-enhanced DSS can be effectively applied in two main contexts: individual patient management (for instance, predicting complications, assisting with diagnosis or estimating treatment responses) and comprehensive population management/screening, which can guide the triage process and facilitate a broader reorganisation of care delivery. DSS aim to aid clinicians by consolidating and interpreting information from various data sources to streamline workflows, ease cognitive burden, and provide clear, actionable insights at the point of care. This may be particularly useful in the context of diabetes, which is often managed by generalist physicians. These physicians may find it challenging to stay updated with complex and changing guidelines, medications and monitoring requirements, whilst simultaneously being faced with short appointment times and high (and growing) patient volumes (see Fig. 4).

Whilst significant academic progress has been made to develop DSS and predictive models using AI, this is not currently matched by investment in the development or implementation of software tools that integrate into the existing digital platforms that are used in common clinical settings. This translational step is necessary to deliver improved care and derive clinical benefit.

### How can AI or DSS be used for diabetes care?

In 2019, Dankwa-Mullan et al conducted a review of publications on AI in diabetes, dividing these into the following categories: (1) predictive population risk stratification (135 publications); (2) clinician DSS (126 publications); (3) automated retinal screening (96 publications); and (4) patient self-management tools (94 publications), mainly focusing on glucose sensors and closed-loop technology [9]. Other reviews have specifically focused on AI applications in a local context (e.g. in India) [10], on detection, diagnosis, and self-management [11], and on diabetes education and management [12]. To further explore uses of AI for diabetes care, in this review, we divide diabetes-associated AI applications into three broad categories, covering both individual and population tools:

1. patient self-management (education and support, and insulin and glucose management);
2. image analysis (retinopathy, other applications); and
3. clinician DSS (complications prediction, diagnostic support, personalised treatment).

#### Patient self-management: education and support

The benefits of education and self-management support in diabetes are well established, but attendance at traditional face-to-face delivery is poor, likely due to inaccessibility, competing priorities and poor motivation [13]. Digital tools to flexibly deliver broad education or point-of-need intervention could be very effective.

AI can support personalisation of educational content and advice, allowing scaled delivery of tailored educational content. Population-level tools exist that apply AI-based approaches to electronic-health-record and home-recorded data, producing data visualisations (e.g. HbA_1c_ trends or target summaries), enabling improved health understanding and patient empowerment [14]. Insights from this data can be presented as personalised recommendations and nudges to encourage healthy lifestyle choices, early identification of problems when they arise and optimal engagement with healthcare services. Digital tools and remote support may be especially advantageous for managing gestational diabetes. Glucose-monitoring notifications and recommendations for medication and lifestyle adjustment may prove highly effective, particularly since women with gestational diabetes are typically young, digitally literate, and motivated to engage with digital tools due to their flexibility and desire to improve self-care to mitigate adverse pregnancy outcomes [12, 15].

The potential to integrate lifestyle data, such as physical activity and nutritional intake, enables more sophisticated automated nudge solutions to encourage activity and behaviour change and provide feedback to the individual [16–20]. Further, this data can enhance glucose and insulin dose calculations. The advent of smartphone applications that can predict nutritional macronutrients from photographs [18], phone accelerometers and wearables to collect activity information, and continuous and flash glucose-monitoring systems for high-frequency automatic glucose tracking is improving the ease and volume of home-recorded real-time data delivery to drive AI advice tools for both patients and clinicians.

Risk prediction of acute and chronic diabetic complications is usually reserved for clinician tools; however, patient-facing tools that demonstrate risk of future complications directly to patients have been developed [21, 22]. Such estimates, when carefully presented as calculators, can help to explain risk subcomponents and act as both motivators for behaviour change and metrics through which goals can be set (and progress towards them tracked). Additionally, novel tools are emerging or being evaluated that incorporate interlinked personalised predictive models and automated e-coaching, many based on behavioural change theories [23].

#### Patient self-management: insulin and glucose management

Numerous commercial AI-driven glycaemic technologies are available today, including glucose-monitoring and insulin-dosing systems that range in complexity and autonomy. Notably, the advent of wearable sensors that enable intermittently scanned or continuous glucose monitoring have transformed day-to-day glucose monitoring. They provide real-time glucose trajectories and trends, feature high and low glucose alarms, and interface with insulin-delivery systems and algorithms allowing autonomous closed-loop insulin administration. In terms of insulin administration, AI-driven systems span a broad spectrum; they extend from basic bolus dose calculations to progressive insulin titration algorithms, right up to highly sophisticated, autonomous continuous insulin infusion closed-loop systems designed for continuous insulin infusion (termed as the 'artificial pancreas'). Many trials have demonstrated that continuous glucose monitoring and closed-loop technologies improve time in target blood glucose range and reduce hypoglycaemic events [20, 24]. Psychosocial outcomes are typically favourable, including reduced diabetes distress and improved sleep, although challenges related to technical issues and dependence on technology persist [25, 26]. For type 2 diabetes specifically, AI-based DSS particularly focus on automated insulin titration recommendations; when combined with clinician support, these systems have been demonstrated to outperform clinician support alone [27]. Machine-learning techniques for predicting hypoglycaemia provide another active area of research. Multiple physiological data sources, such as blood glucose data, electronic health records and electrocardiography data, are being utilised to optimise prediction [28]. This approach has substantial potential to lay the groundwork for future tools in diabetes management.

#### Image analysis: retinopathy

Retinal image analysis for the detection of diabetic retinopathy is an area that is ripe for exploitation through AI, with the potential to reduce the human resource requirement for retinopathy screening and grading [29]. Many autonomous AI systems for diabetic retinopathy detection have been or are now being developed and, in some cases, are built into commercially available systems displaying safety and efficacy, resulting in Food and Drug Administration (FDA) approval [30–32]. Auto-graders for low-risk eye screening have been used at scale as part of national programmes in the UK for many years, with acceptable safety and efficiency savings [33]. Further, the characteristics of retinal imagery, both alone and in combination with genetic data, have shown it to have utility as a biomarker for the prediction of wider cardiovascular risk as well as complications related to other organs (owing to shared underlying pathophysiology) [34, 35].

#### Image analysis: other applications

Beyond the eye, many other diabetes applications for image analysis exist. Uses relating to screening for diabetic foot disease and associated wound analysis is one such example [36]. Understanding the evolution of wounds is a common clinical challenge, particularly when documentation is limited to text-based descriptions. Data-driven systems can improve foot monitoring quality by simply providing a repository for home- or clinician-recorded photographic data in a standardised format [37]. Once such data are centralised, AI-based tools can analyse imagery to screen for foot problems, or sequentially track active foot wounds through monitoring of surface features (e.g. area, colour) providing relevant alerts in the event of clinical deterioration. Interestingly, deep-learning approaches have recently been used to uncover new uses for electronic-health-record imaging data collected for other purposes, as demonstrated by a recent study presenting a model that predicts type 2 diabetes diagnosis from chest radiographs [38]. Explainability analysis suggested distribution of adiposity (particularly mediastinal lipomatosis) as a predictive driver, yielding biological insights in addition to predictive screening potential.

#### Clinician decision support: complications prediction

One of the most prolific uses of big healthcare data is the prediction of future health states. For diabetes, this usually relates to prediction of short- and long-term complications, such as hospitalisation, low and high blood glucose events (including ketoacidosis), and macro- and microvascular complications, such as cardiovascular disease, and eye, feet and kidney complications [39]. Some of the earliest cardiovascular risk-predictor tools came from the Framingham studies [40] and, later, the UK Prospective Diabetes Study [41]. Since then, many models and tools have been developed that focus on specific complications, such as retinopathy (to determine personalised screening intervals) [42], or hypoglycaemia prediction [43]. Prevention of foot complications, such as ulcers and amputations, could translate to significant health and social care cost savings if linked to early prevention interventions. Simple rule-based foot risk calculators (such as that developed by the Scottish Diabetes Foot Action Group) have been automated through the Scottish Care Information (SCI)-diabetes platform, leading to personalised screening follow-up and self-management advice [44].

#### Clinician decision support: diagnostic support

Clinician DSS in diabetes can also assist with risk prediction and diagnosis of diabetes [45], and subtyping of diabetes into type 1 and type 2 diabetes and rarer but well-established monogenic subtypes [46]. Type 2 diabetes, however, is a highly heterogeneous condition, and further subtyping into distinct groups based on phenotypic and polygenic clustering may help to predict prognoses and preferential treatment responses; this complexity lends itself well to an AI-driven DSS approach [47].

#### Clinician decision support: personalised treatment and prescribing for type 2 diabetes

As the number of pharmacological options for lowering glucose levels in type 2 diabetes increases, prescription decisions become more challenging, particularly for generalist prescribers. In addition to glycaemic benefits, it is increasingly important to consider the longer-term cardiovascular benefits of medication, as reflected in recent European and US guidelines [48]. Personalised prescribing could improve outcomes, rationalise choice of medications and minimise side effects [2, 49]. Routinely collected electronic-health-record data alone can help to predict patient medication response [50]. Pharmacogenomics is a growing area of investigation; we know that genetic variation plays a contributory role in interindividual differences in response to multiple drugs, potentially through modulation of narrow biological drug-action pathways [51]. As links between genetic variants and drug response and intolerance phenotypes grow, incorporation of genetic data in treatment-response prediction models will become increasingly important. Increasing the volume, depth and modality of data inputs will likely improve accuracy and application of AI tools; emerging DSS systems are trying to capitalise on such multimodal inputs [52].

### Do diabetes-related AI and DSS work?

A 2020 meta-analysis of controlled trials showed that, overall, data-driven DSS can improve health outcomes, delivering, on average, small mean improvements in outcomes with wide variation (mirroring the interventions and clinical settings assessed) [53]. Similarly, a 2018 review of 70 inpatient DSS studies (of which 14 pertained to blood glucose management) found that 70% had beneficial outcomes (including reduced mortality and reduced life-threatening events), 29% found no benefit and only one study showed harm (increased hypoglycaemia) [54]. Overall, the evidence for improved outcomes and impact relating to the use of AI and linked DSS in diabetes (particularly beyond glucose control or insulin management) is still emerging, and highly context and system dependent.

A 2019 review of systematic reviews for diabetes-related clinical DSS that comprised varied interventions concluded that they ‘improved the quality of diabetes care by inconsistently improving processes of care or patient outcomes’ (this was observed in 85% and 31% of the studies assessed, respectively) and determined that providing alerts, reminders or feedback was most likely to have an impact on diabetes care [55]*.* The authors noted that methodology was poorly reported, limiting confidence in the available evidence. A 2020 review of diabetes-related DSS concluded that ‘intelligent technical reforms have produced better glyc[a]emic control with reductions in fasting and postprandial glucose levels, glucose excursions, and glycosylated h[a]emoglobin’ [56]. Others have shown a reduction in the time devoted by clinicians to patients and in face-to-face visits per patient [3]. A 2013 meta-analysis by Jeffery et al showed that clinician DSS in diabetes management may marginally improve clinical outcomes, but confidence in the evidence was low because of bias and study heterogeneity [57]. O'Connor and colleagues found similar limitations of the evidence base but suggested that systems that support patient communications and integrate patient-record and remote device information in clinical decision algorithms and interfaces are more likely to be effective [58]. This is reflected in current development trends; an example of a successful health-record integrated DSS is described by Conway et al, with significant associated improvements in clinical outcomes and clinician adherence to screening recommendations compared with matched control participants [59].

Evidence syntheses are clearly important to understand DSS as a concept and identify techniques or domains in which efficacy seems most likely. However, as reviews identify, AI technologies are incredibly diverse in their user interface, experience, features, sophistication and reliability [3, 7, 9–12, 15]. Such diversity undermines attempts to meta-analyse efficacy data; poor quality, ineffective DSS should not detract from effective and adequately evidenced interventions that could yield benefit. We therefore caution drawing conclusions based on what the average DSS system might achieve, as only the best should be clinically implemented. Selecting the best DSS, those that warrant incorporation into daily clinical practice, remains a challenge, and existing evidence is complicated by the quantity of low-quality non-randomised trials at high risk of bias [54]. Further, the existing evidence shows that many diabetes-related DSS do not translate into improved clinical outcomes, particularly regarding longer-term clinical outcomes, which are seldom assessed. Where other systems show benefit, typically modest improvements in glycaemic or metabolic parameters are observed. Notably, DSS systems need not work in isolation; these effects could be compounded by implementation of numerous DSS seeking to influence behaviour across multiple domains.

There are many important priorities that future research on DSS should address. Current evidence shows that most DSS remain rule based [54]; more complex AI-based DSS may impact outcomes differently. Unpicking variations in outcomes between rule- and more complex AI-based DSS should be a focus for future evidence synthesis research. Other research priorities should include a focus on collecting longer-term follow-up data, including quality-of-life outcomes. Qualitative studies to better understand how DSS systems could (positively or negatively) disrupt existing workflows and integrate with existing digital platforms (e.g. electronic health records) would help guide future best practices. A diversity of outcomes is also important to assess. For clinicians, a DSS system that helps achieve screening targets or improves consultation efficiency may be very worthwhile, although such outcomes are not typically reported in existing studies. As many systems aim to improve efficiency at a population scale in a low-cost manner, it is important to incorporate economic analysis into future research.

### Challenges relating to the adoption of AI and data-driven technologies for diabetes care

As discussed, AI and data-driven technologies offer the potential to complement and transform diabetes-care delivery. However, several barriers must be overcome for effective adoption and implementation. These include challenges related to data accessibility, sharing and standardisation, regulatory compliance and safety considerations, equity aspects, and the attitudes of both clinicians and patients.

#### Data availability and flow

For data-driven and AI technologies to successfully leverage healthcare data, access to data in a usable format is essential. This necessitates the basic digitisation of healthcare records and, although progress has been made, substantial variability in digital maturity of healthcare organisations exists between and within European countries. For example, 69% of clinicians practising in Italy reported usage of an electronic health record compared with 77% in Germany, 87% in the UK and 97% in the Netherlands [60]. Clearly, ongoing investment in basic digital infrastructure is still required. Further, the way in which data are stored and exchanged digitally is important when considering how data-driven technologies may be developed or integrated. Healthcare datasets typically exist, containing non-standardised data (with varying structures/naming conventions), siloed in numerous incompatible record systems within and across countries. This extreme diversity makes efficient sharing and utilisation of information difficult, and system crosstalk between internal, external or novel systems challenging. This lack of digital interoperability limits data sharing between people, care settings and organisations, posing a significant barrier to development or scaled integration of innovative technologies.

Solutions to address such issues are complex and require significant investment. The National Health Service (NHS) England has recently begun formal procurement for a £480 million federated data platform that aims to centralise data to facilitate sharing [61] and has developed national diabetes data standards [62]. The European Commission plan to digitise all medical records in the European Union (EU) by 2025 through creation of the European Health Data Space [63]. Other European initiatives focusing on the development of data dictionaries, standards and core outcome sets have potential to improve data sharing, although success is dependent on consistent adoption [64, 65]. Machine-learning approaches can also contribute by aiding in the identification of common features within datasets, which can assist in data harmonisation. Additionally, federated-learning approaches allow the training of models using multiple discrete datasets located in different physical locations. In this decentralised method, models are trained locally on each dataset and the learned parameters are subsequently aggregated to form a global model. This ensures data privacy and reduces the need for data centralisation, whilst still enabling researchers to benefit from the insights gathered across diverse data sources.

#### Fairness and health equity

AI technologies have the potential to revolutionise diabetes-associated decision-making in resource-limited settings by providing accurate and timely decision support or facilitating low-cost population health surveillance. However, such opportunity may be undermined by the limited generalisability of these technologies outside of the datasets and populations from which they are trained [66]. Despite diabetes disproportionally affecting ethnically minoritised and socioeconomically disadvantaged groups [67], AI training datasets are often inadequately diverse. Globally, datasets predominantly focus on cohorts from high-income countries, with a preponderance of individuals of European ancestry [66]. Consequently, applications can demonstrate learned bias or be limited to use within specific groups; AI-associated exacerbation of racial or ethnic health inequality should not be underestimated. To mitigate potential widening of health disparities, it is crucial to establish data infrastructures in data-poor regions to proactively improve the diversity of training datasets; diverse stakeholder groups should be consulted prior to development taking place.

Delivering data-driven tools directly to people with diabetes could result in widening health inequalities. Digital exclusion can occur through poor digital literacy or inability to use, access or afford technology or internet access. In the UK, an estimated 5 million people are not connected to the internet and only around half of the global population are online [68]. Many drivers of digital exclusion exist that compound one another (e.g. ethnicity, deprivation, age, education and sex). Type 2 diabetes disproportionately affects older adults, a population in which digital skills are limited and musculoskeletal, hearing, visual and cognitive impairments contribute to reduced access [69]. As the inevitable trend towards digitally enabled healthcare continues, strategies (e.g. user co-design, combatting inherent biases) to improve access for older people are necessary.

#### Patient and clinician attitudes

The attitudes of both patients and clinicians in relation to the use of AI and data-driven technologies in diabetes care is central to their adoption. A recent systematic review of patient attitudes towards clinical AI found general positivity and willingness to engage but noted several reservations [70]. Patients sought proof of effectiveness and understanding of the exact application of AI technology. Concerns included dependence on technology, and the risk of depersonalisation, particularly relating to use of chatbots. As such, patients highlighted their desire for clinician involvement and oversight. Broadly, clinicians embrace the synthesis of information, improved diagnostic accuracy and reduced administrative burden that AI can bring [71]. However, concerns focused on liability for AI-associated errors, privacy and confidentiality, poor understanding and AI-related training, and potential for erosion of patient contact.

#### Safety, security and regulatory considerations

Ensuring clinical safety and data security is essential for successful implementation of AI and data-driven tools in diabetes care. Recently, the public release of large language models, such as Generative Pre-trained Transformer (GPT)-4 (ChatGPT) has led to concerns regarding the potential risks and safety of the unsupervised healthcare advice generated by these models [72]. The regulatory landscape in Europe and the USA has rapidly evolved over recent years to accommodate the shift from regulation of largely physical medical devices to software as a medical device [73]. This has been challenging, particularly given the demand from software companies and the limited capacity of regulatory consultants and notified bodies to review and support regulatory submissions. Brexit has further created a disparity between UK and EU regulatory requirements, at least in the short term. In a 2020 report, dozens of FDA-cleared medical devices that use AI/machine-learning technology were reported. Most of these approvals are linked to radiology, cardiology and oncology, and only three AI-based medical devices were related to diabetes management [74]. Clinical approval bodies, such as the National Institute for Health and Care Excellence in the UK, have been slow to develop scalable pathways for approval of AI-driven tools and software more generally. A recent Regulatory Horizons Council UK government report expressed concern regarding a lack of understanding to ensure effectiveness of these technologies, and how best to detect, analyse, report and action errors and potential harms arising from usage [75]. Many devices go through approvals with minimal evidence as to their real-world safety and there is little consensus as to how this safety should be assured. The report recommends significant government investment in AI regulation, and the development of transparent clear frameworks, processes and leadership.

## Conclusion

Although the development of AI-driven functionality in healthcare is expanding rapidly, AI-enabled DSS largely remain in their infancy. Globally, the market for AI health technologies is expected to grow at a compound annual growth rate of 38.4% from 2022 to 2030, reaching 208 billion US dollars by 2030 [76]. In parallel, future diabetes projections look bleak; forecasts estimate that by 2050, 1.31 billion people will be living with diabetes [77]. Recent decades have seen major cultural shifts in eating habits and activity levels that have catalysed an obesity crisis and, in 2021, 96% of diabetes cases were reported as being type 2 diabetes. It is evident that a comprehensive approach is necessary and, although AI-based tools alone will be no panacea, their benefits must not be ignored. Such tools can be delivered at low cost and scaled throughout a population or clinical workforce to deliver significant benefit. The anticipated increase in data availability, coupled with enhanced data access, is likely to yield superior predictive abilities, utility, adoption and widespread clinical impact. Yet, the practical, timely and ethical integration of these tools into existing clinical scenarios continues to pose a challenge. Nevertheless, the momentum is unmistakably shifting, and all stakeholders—citizens, public institutions and private organisations—must swiftly adapt to both reap the benefits and reduce the risk that our digitised and AI-enabled new world could bring.

### Supplementary Information

Below is the link to the electronic supplementary material.

Supplementary file1 (PPTX 663 KB)

## Abbreviations

AI: Artificial intelligence
DSS: Decision support system
EU: European Union
FDA: Food and Drug Administration
GPT: Generative Pre-trained Transformer
