# Supplementary material for: Diabetes and artificial intelligence beyond the closed loop: a review of the landscape, promise and challenges
Source: Diabetologia. 2023 Nov 18;67(2):223–35. doi: 10.1007/s00125-023-06038-8 (PMC10789841; doi:10.1007/s00125-023-06038-8)
Supplement: Supplementary file 1 — Supplementary file1 (PPTX 663 KB) [file 125_2023_6038_MOESM1_ESM.pptx]

## Slide 1
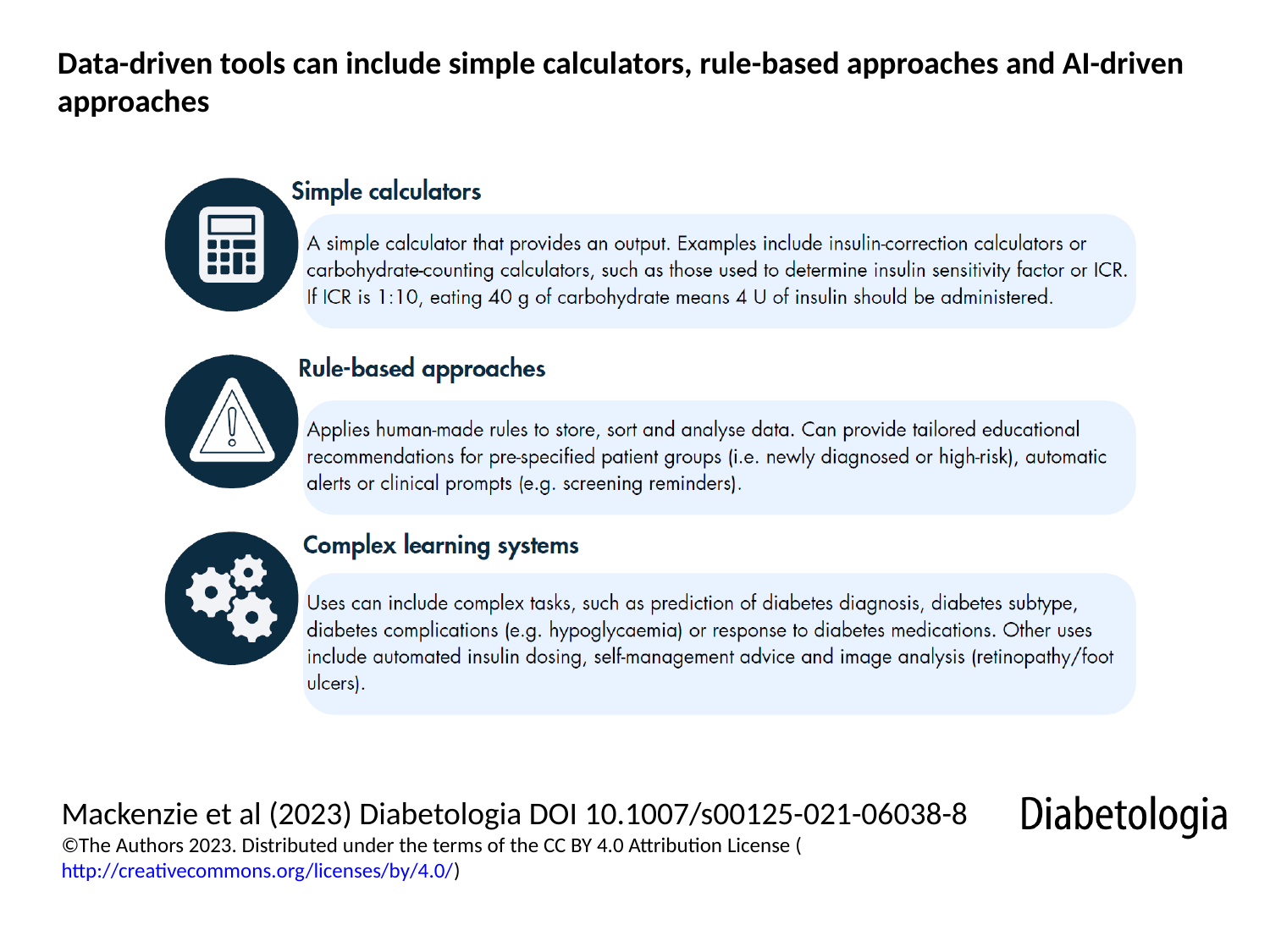

Data-driven tools can include simple calculators, rule-based approaches and AI-driven approaches
Mackenzie et al (2023) Diabetologia DOI 10.1007/s00125-021-06038-8
©The Authors 2023. Distributed under the terms of the CC BY 4.0 Attribution License (http://creativecommons.org/licenses/by/4.0/)

## Slide 2
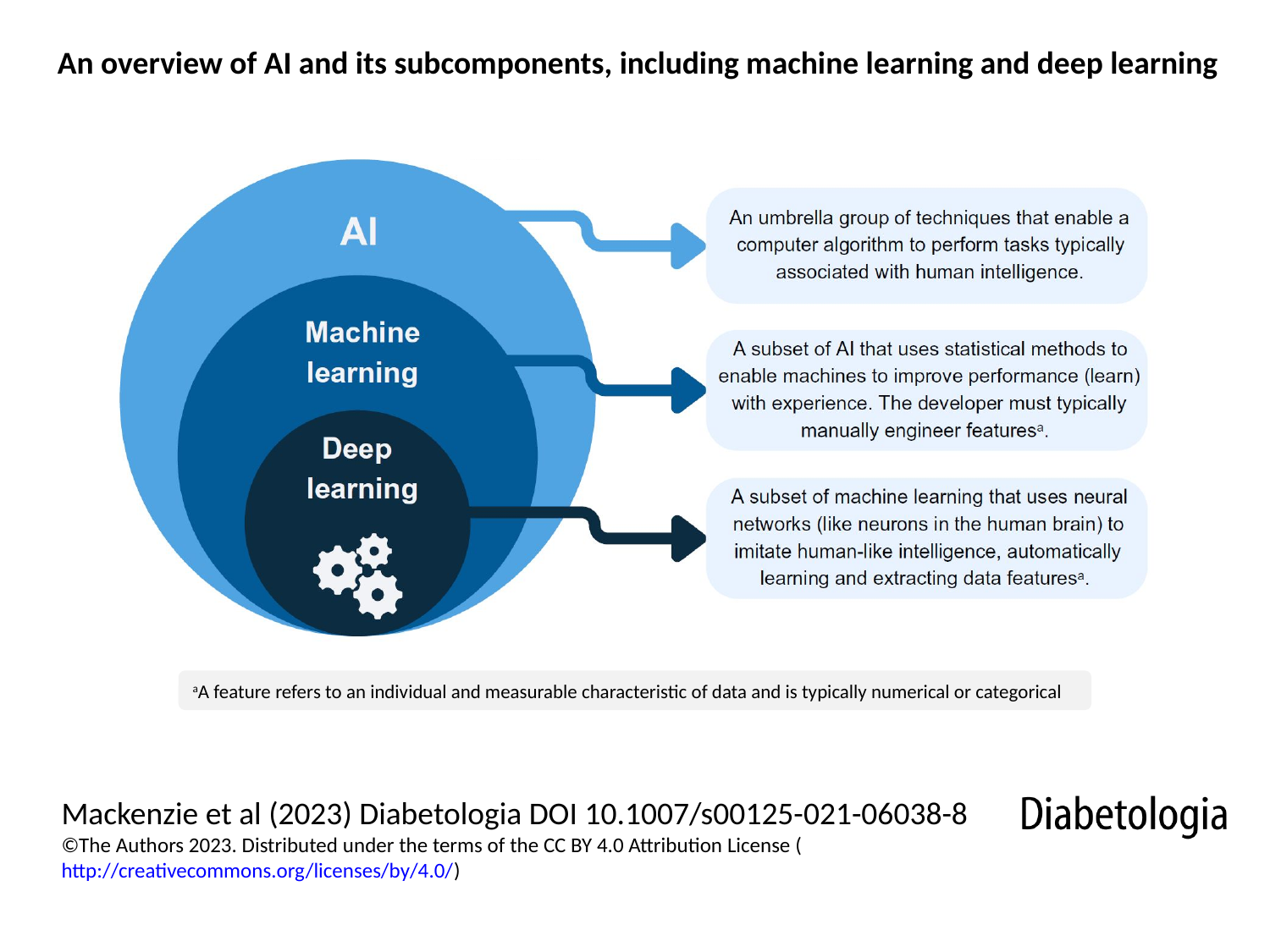

An overview of AI and its subcomponents, including machine learning and deep learning
aA feature refers to an individual and measurable characteristic of data and is typically numerical or categorical
Mackenzie et al (2023) Diabetologia DOI 10.1007/s00125-021-06038-8
©The Authors 2023. Distributed under the terms of the CC BY 4.0 Attribution License (http://creativecommons.org/licenses/by/4.0/)

## Slide 3
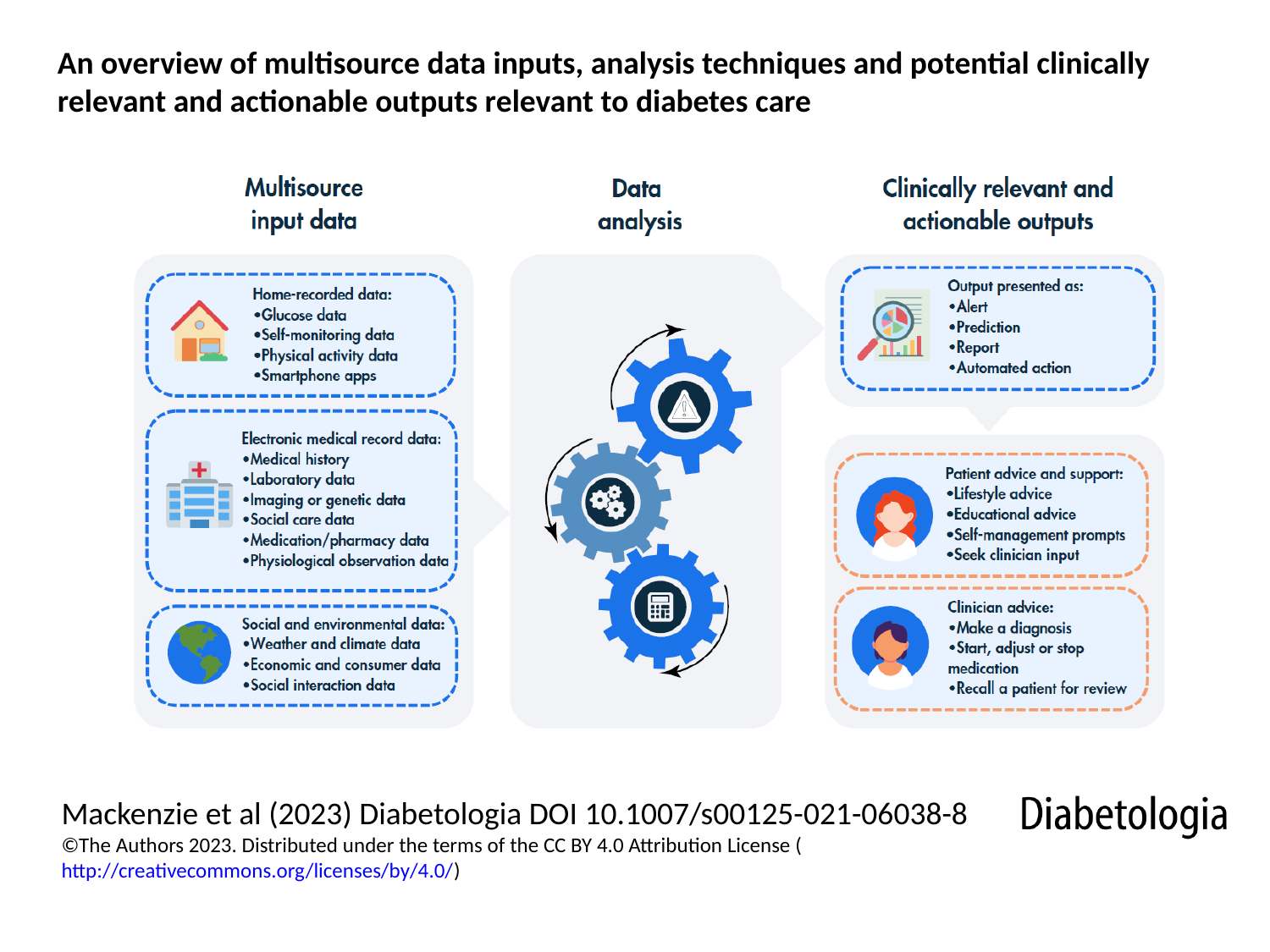

An overview of multisource data inputs, analysis techniques and potential clinically relevant and actionable outputs relevant to diabetes care
Mackenzie et al (2023) Diabetologia DOI 10.1007/s00125-021-06038-8
©The Authors 2023. Distributed under the terms of the CC BY 4.0 Attribution License (http://creativecommons.org/licenses/by/4.0/)

## Slide 4
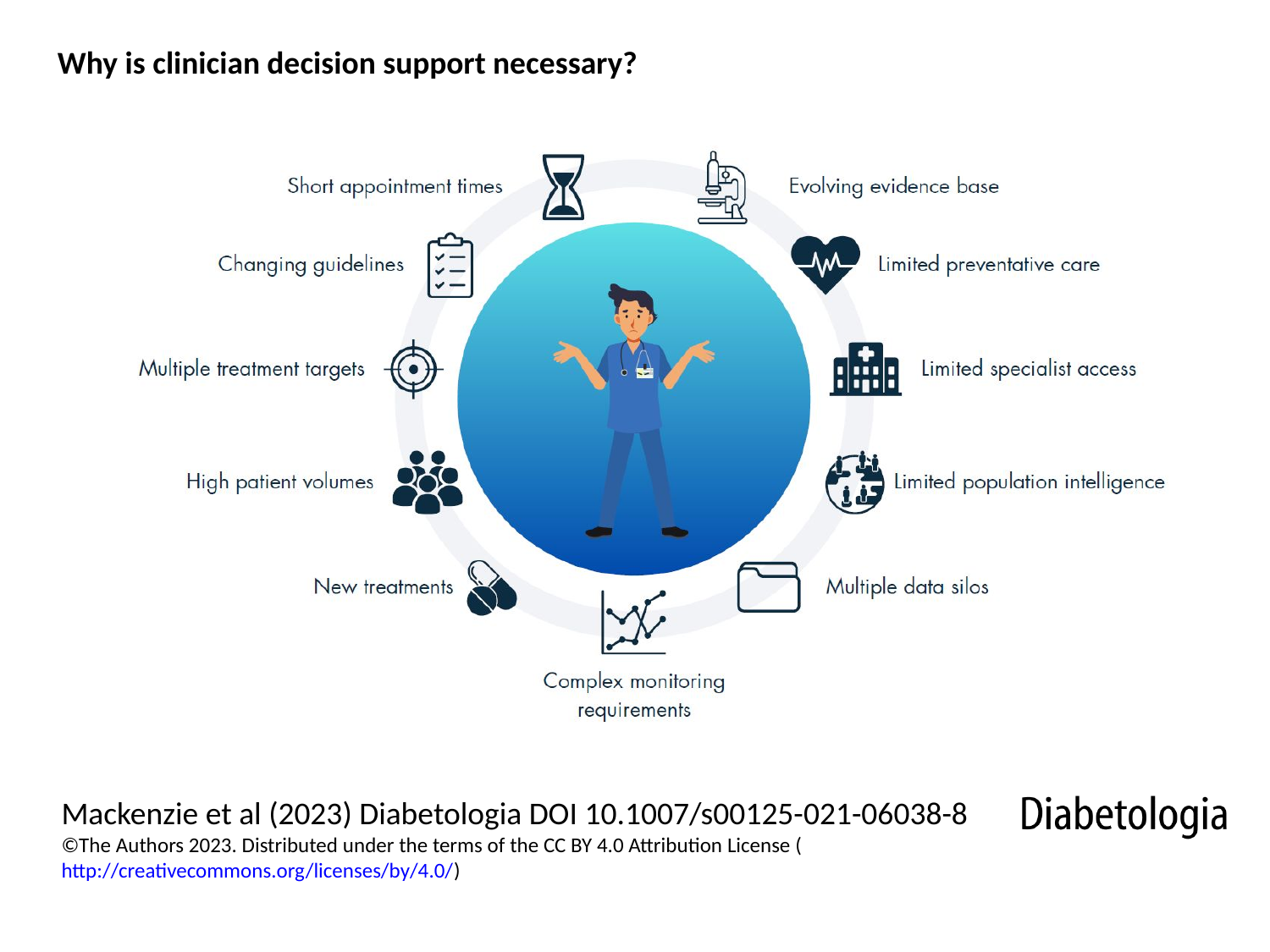

Why is clinician decision support necessary?
Mackenzie et al (2023) Diabetologia DOI 10.1007/s00125-021-06038-8
©The Authors 2023. Distributed under the terms of the CC BY 4.0 Attribution License (http://creativecommons.org/licenses/by/4.0/)
